# Phosphoprotein Gene of Wild-Type Rabies Virus Plays a Role in Limiting Viral Pathogenicity and Lowering the Enhancement of BBB Permeability

**DOI:** 10.3389/fmicb.2020.00109

**Published:** 2020-02-20

**Authors:** Teng Long, Boyue Zhang, Ruqi Fan, Yuting Wu, Meijun Mo, Jun Luo, Yiran Chang, Qin Tian, Mingzhu Mei, He Jiang, Yongwen Luo, Xiaofeng Guo

**Affiliations:** College of Veterinary Medicine, South China Agricultural University, Guangzhou, China

**Keywords:** HEP-Flury, GD-SH-01, phosphoprotein, blood–brain barrier, viral replication

## Abstract

Enhancement of blood–brain barrier (BBB) permeability is necessary for clearing virus in the central nervous system (CNS). It has been reported that only laboratory-attenuated rabies virus (RABV) induces inflammatory response to lead BBB transient breakdown rather than wild-type (wt) strains. As a component of ribonucleoprotein (RNP), phosphoprotein (P) of RABV plays a key role in viral replication and pathogenicity. To our knowledge, the function of RABV P gene during RABV invasion was unclear so far. In order to determine the role of RABV P gene during RABV infection, we evaluated the BBB permeability *in vivo* after infection with wt RABV strain (GD-SH-01), a lab-attenuated RABV strain (HEP-Flury), and a chimeric RABV strain (rHEP-SH-P) whose P gene cloned from GD-SH-01 was expressed in the genomic backbone of HEP-Flury. We found that rHEP-SH-P caused less enhancement of BBB permeability and was less pathogenic to adult mice than GD-SH-01 and HEP-Flury. In an effort to investigate the mechanism, we found that the replication of rHEP-SH-P has been limited due to the suppressed P protein expression and induced less response to maintain BBB integrity. Our data indicated that the P gene of wt RABV was a potential determinant in hampering viral replication *in vivo*, which kept BBB integrity. These findings provided an important foundation for understanding the viral invasion and development of novel vaccine.

## Introduction

Blood–brain barrier is a physical barrier between the CNS and peripheral environment, which impedes the entrance of VNA from peripheral blood into CNS to eliminate virus when the host was infected with lethal RABV. To better understand the genetic function of RABV, we compared the BBB permeability *in vivo* among grouped mice infected with GD-SH-01, HEP-Flury, and rHEP-SH-P, and we found the RABV P contributes negatively to the enhancement of BBB permeability by limiting viral replication and induced less inflammatory response. This study demonstrates that the P gene of RABV plays a potential determinant in hampering the viral proliferation to realize immune escape during the infection course, which gives a framework to understand the genetic function better and develop more efficient therapeutic strategies against rabies.

Rabies virus (RABV) is a non-segmented negative-stranded RNA virus in genus *Lyssavirus* within the *Rhabdoviridae* family (Schnell et al., 2010). It causes an acute and fatal zoonotic disease, where there is no cure once clinical symptoms develop and is responsible for more than 55,000 human deaths every year (Fu, 1997; Jackson, 2003). Despite the fact that most dog rabies has been eliminated in developed countries through vaccination (Blanton et al., 2006), majority of the human rabies cases occurred in poor areas where dog rabies remained prevalent (Fu, 1997).

While only mild inflammation was observed in the central nervous system (CNS) of patients infected with the lethal RABV at early stage (Wang et al., 2005; Kuang et al., 2009), however, laboratory-attenuated virus induced severe inflammatory response and dendritic cell activation (Li et al., 2008; Chai et al., 2014). To understand the mechanism of virus clearing, MCP-1, an inflammatory molecule, was administrated from peripheral vessels to enhance the blood–brain barrier (BBB) permeability, so that the virus-neutralizing antibody (VNA) entered CNS to eliminate RABV (Huang et al., 2014). Therefore, the enhancement of BBB permeability is necessary for the entrance of VNA from the periphery into CNS. It has been reported that the failure to enhance the BBB permeability in beginning stage causes the lethal outcome after infection with wild-type (wt) RABV (Roy et al., 2007); only the laboratory-attenuated RABV infection induces high level of cytokines/chemokines to decrease tight-junction (TJ) protein expression and cause the transient enhancement of BBB permeability to avoid development of rabies. RABV-induced inflammatory cytokines/chemokines are the key mediator of BBB permeability enhancement; for example, interferon gamma-induced protein 10 (CXCL10) mediates the recruitment and differentiation of CD4^+^ T cells, which results in BBB breakdown (Chai et al., 2015; Gnanadurai and Fu, 2016).

Although the inflammatory response is necessary to protect hosts from RABV infection, severe antiviral immune mechanisms also contribute to rabies pathogenesis (Hooper, 2005). Previous studies suggested that although BBB permeability was enhanced and aided T cell infiltration, neural damages caused by severe inflammation were observed in infected mice succumbed to a high dose of attenuated virus (Morimoto et al., 1998; Chai et al., 2014). Therefore, lethal rabies can be prevented only if mild rather than severe inflammation develops during viral infection, especially in CNS.

Phosphoprotein (P) gene of RABV plays a major role in the neuro-invasiveness by mediating infection of peripheral nerves (Yamaoka et al., 2013) and serves as an interferon antagonist resulting in immune escape (Ito et al., 2010). In our previous study, we rearranged the P gene of RABV and found that low level of P gene expression suppressed N gene and result in pathogenicity attenuation (Mei et al., 2017), which suggested that the viral replication and pathogenicity were consistent with P gene expression indirectly. However, the role of P protein in the course of RABV invasion *in vivo* was unclear so far. In an effort to determine the role of RABV P protein during infection, we evaluated the BBB permeability *in vivo* after infection with wt RABV strain (GD-SH-01), laboratory-attenuated RABV strain (HEP-Flury), and a chimeric RABV strain (rHEP-SH-P) that was rescued in our laboratory previously (Tian et al., 2017b). We observed less permeable BBB in the rHEP-SH-P group than that of parent strains. To investigate the mechanism, we found that the proliferative capacity of rHEP-SH-P *in vivo* was limited due to the suppressed P protein expression compared to HEP-Flury and GD-SH-01, which further induced weaker inflammatory response and pathogenicity to keep BBB integrity.

## Materials and Methods

### Virus, Antibody, and Animals

Three RABVs were used in this study, including GD-SH-01, HEP-Flury, and rHEP-SH-P. GD-SH-01 is a wt RABV strain that was isolated from the brain tissue of rabid pig in our laboratory and is phylogenetically close to canine RABV (Luo et al., 2012, 2013), HEP-Flury, a laboratory-attenuated high egg passage (HEP) RABV strain, which is preserved in our laboratory, and rHEP-SH-P, which is a chimeric strain whose P gene was adopted from GD-SH-01 and expressed in the backbone of HEP-Flury genome (Tian et al., 2017a). Rabbit polyclonal anti-Claudin-5 antibody, rabbit monoclonal anti-Occludin antibody, and rabbit polyclonal anti-CD3 antibody were purchased from Abcam (Cambridge, MA, United States). Female Kunming (KM) mice (6–8 weeks old) were purchased from the Center for Laboratory Animal Science at the Southern Medical University (Guangzhou, China). Mice were housed in the Laboratory Animal Center of South China Agricultural University.

### Virus Infection and Pathogenicity

Eight 6- to 8-week-old KM mice per group were infected intranasally(i.n.) with 10^5^ focus-forming units (FFU) of GD-SH-01, HEP-Flury, or rHEP-SH-P, and the same volume of RPMI 1640 medium (Gibco, MA, United States) for mock infection. For pathogenicity, mice were daily weighed for successive 15 days, and the symptoms in mice were classified into five grades (Yamaoka et al., 2013): (a) normal, (b) body weight loss (5% reduction from original body weight), (c) mild neurological signs (stagger or gait abnormality of a unilateral hind limb), (d) severe neurological signs (such as gait abnormality of bilateral hind limbs), and (e) death. All animal experiments were carried out under protocols in compliance with national standard Laboratory Animal Requirements of Environment and Housing Facilities (CALAS, GB14925-2001), following the guidelines of the Humane Treatment of Laboratory Animals. Records of weight and clinical signs can be available in Supplementary Tables S2, S3 respectively.

### Measurement of BBB Permeability

Blood–brain barrier permeability was assessed using sodium fluorescein (NaF) as a tracer molecule according to a previously described technique (Phares et al., 2007; Luo et al., 2018). Mice were administered intravenously with 100 μl of 10% NaF in phosphate-buffered saline (PBS), and after 10 min to allow circulation of NaF, peripheral blood was collected, and the mice were transcardially perfused with PBS after anesthesia. Cerebrum and cerebellum were separated after brain tissues were harvested. The serum was mixed with an equal volume of 15% trichloroacetic acid (TCA) and was centrifuged at 10,000 rpm for 10 min; the supernatant was then collected and mixed with 5 M NaOH to 150 μl. Homogenized brain samples in 7.5% TCA were centrifuged at 10,000 rpm for 10 min, and the harvested supernatant was mixed with 5 M NaOH to 150 μl. The fluorescein of brain and serum samples was measured using BioTek spectrophotometers (BioTek, Winooski, VT, United States) with excitation at 485 and emission at 530 nm. NaF uptake into brain tissue was regarded as the BBB permeability, which was normalized with fluorescence from peripheral blood; the result was expressed as (μg fluorescence brain tissue/mg tissue)/(μg fluorescence sera/ml blood).

### Western Blot Analysis

Three independent mice from each of the infection groups were perfused intracardially with PBS after anesthesia at the indicated time point. Brain tissues were harvested and homogenized on ice using RIPA buffer (Beyotime, Shanghai, China). The lysates were centrifuged at 12,000 rpm for 30 min, and the supernatants were harvested or stored at −80°C. Proteins were separated by 12–15% SDS-PAGE, and target proteins were electroblotted onto PVDF membranes and incubated with antibody against Occludin, Claudin-5, or β-actin. After a second incubation with goat anti-mouse or goat anti-rabbit antibody conjugated with HRP, the membranes were stained with BeyoECL Plus A and B (Beyotime, Shanghai, China) according to the manufacturer’s instructions. Protein fingerprints were shown using Fine-do x6 (Tanon, Shanghai, China). The expression levels of TJ proteins were analyzed by quantifying integrated optical densities (IODs) using Image J v1.51 and normalized by house-keeping gene β-actin.

### Real-Time Quantitative RT-PCR

Three independent brain tissues were removed from infected mice at the indicated time point. Homogenized brain samples were lysed using Trizol (Magen, Guangzhou, China) following the manufacturer’s instructions. Total RNA was extracted using the HiPure Universal RNA Kit (Magen, Guangzhou, China) and used for first cDNA synthesis (Vazyme, Nanjing, China) according to the manufacturer’s protocol. Each reaction was carried out in triplicate using SYBR Green Master Mix (Vazyme, Nanjing, China) following the manufacturer’s instructions. Quantitative real-time RT-PCR (qRT-PCR) was carried out using a CFX96 Real-Time System (Bio-Rad, Hercules, CA, United States). The nine cytokines and chemokines including CXCL10, CXCL9, CCL5, CCL3, interleukin (IL)-6, IL-17, tumor necrosis factor alpha (TNF-α), IFN-γ, and ICAM-1 were analyzed. The RABV structural gene (N, P, M, G, and L) mRNA level was examined using qRT-PCR. All the expression levels of target genes were normalized to a reference gene, glyceraldehyde-3-phosphate dehydrogenase (GAPDH). The primer pairs used in this study are displayed in Supplementary Table S1.

### Viral Growth *in vivo* and Titration

To determine the viral growth *in vivo* during infection, three mice per group were i.n. infected with 10^5^ FFU HEP-Flury, rHEP-SH-P, or GD-SH-01, respectively, and the same volume of RPMI 1640 for mock infection. The harvested brains of infected mice were homogenized with RPMI 1640 and centrifuged in maximum speed, the neuroblastoma (NA) cells seeded in 96-well plates were inoculated with serial 10-fold dilutions of supernatant and incubated at 37°C for 4 days. The culture medium was discarded, and the cells were fixed with 80% acetone for 30 min at −20°C. The cells were washed three times with PBS and stained with fluorescein isothiocyanate (FITC)-labeled anti-RABV N antibodies (Fujirabio), and the positive foci were counted under a fluorescence microscope (AMG, United States); the virus titers were calculated as FFU/ml using Carber’s method. Moreover, the levels of RABV N mRNA and viral genomic RNA (gRNA) were quantitated as described previously (Wang et al., 2014; Tian et al., 2017a).

### Histopathology and Immunohistochemistry

For histopathology and immunohistochemistry (IHC), anesthetized mice were perfused intracardially with PBS followed by 4% paraformaldehyde as described previously (Yan et al., 2001; Chai et al., 2014). The infected brains were removed and paraffin embedded for coronal sections (thickness, 3–4 μm). The sections were stained with hematoxylin and eosin (HE) for histopathology. For IHC, the slides were de-paraffinized and rehydrated in xylene and ethanol. After retrieval in citrate buffer (pH 6.0; Novocastra, United Kingdom), the sections were blocked with goat serum and incubated with primary antibodies against CD3 in 4°C for 12 h, and then incubated with secondary antibodies after washing three times with PBS. Diaminobenzidine (DAB) (Dako, Copenhagen, Denmark) was utilized for color development. For quantification, the slides were observed without pre-knowledge of groups under 400× magnification using an Olympus BX43 (Tokyo, Japan) microscope and photographed using Canon (Tokyo, Japan) EOS600D digital camera, and the CD3-positive cells within the whole view under the microscope were calculated as the count of the DAB signals (Chai et al., 2014; Luo et al., 2018).

## Results

### The Chimeric RABV Strain Causes Less Enhancement of BBB Than Parent Strains

**FIGURE 1:**
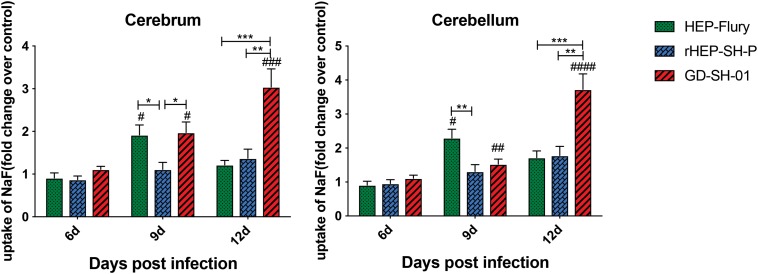
NaF uptake indicating BBB permeability. At 6, 9, or 12 dpi, BBB permeability was assessed by NaF uptake in cerebrum or cerebellum. The statistical analyses were performed using Student’s *t*-test for infected mice and mock group, and one-way ANOVA among infected groups. Significant differences between infected mice and mock infection group were denoted by ^#^*p* < 0.05; ^##^*p* < 0.01; ^###^*p* < 0.001; ^####^*p* < 0.0001. Significant differences between experimental groups were denoted by *p < 0.05; **p < 0.01; ***p < 0.001. Data are means ± SEM of results from three independent experiments.

**FIGURE 2:**
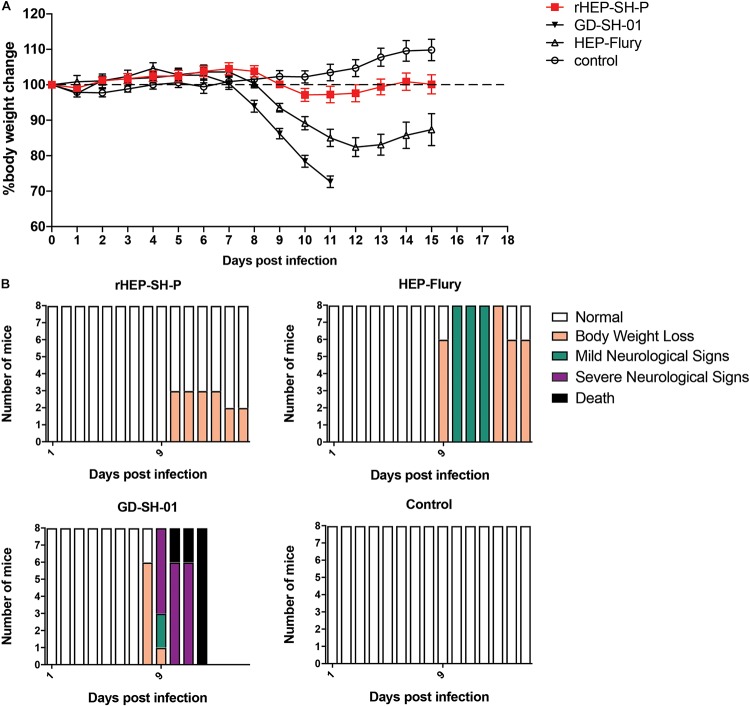
Viral pathogenicity after the grouped mice were intranasally infected with 10^5^ FFU of HEP-Flury, GD-SH-01, or rHEP-SH-P, and culture medium for control. **(A)** Infected mice were weighed for successive 15 days for body weight change. **(B)** Clinical signs of infected mice were recorded for 15 days and classified to five grades: (a) normal, (b) body weight loss (5% reduction from original body weight), (c) mild neurological signs (stagger or gait abnormality of a unilateral hind limb), (d) severe neurological signs (such as gait abnormality of bilateral hind limbs), and (e) death.

In order to evaluate the BBB permeability after infection with HEP-Flury, rHEP-SH-P, and GD-SH-01, the leakage of NaF from peripheral circulation into CNS was measured in cerebra and cerebella of infected mice at 6, 9, and 12 days post infection (dpi). As shown in Figure 1, the BBB was found to be not significantly permeable among three groups in both cerebra and cerebella at 6 dpi, while by 9 dpi, the BBB permeability of mice infected with HEP-Flury and GD-SH-01 was observed to be much greater (around twofold increase of NaF uptake) than in those infected with rHEP-SH-01 in cerebra. Although no significant difference between rHEP-SH-P and GD-SH-01 was detected in cerebella BBB permeability changes at 9 dpi, GD-SH-01 caused great increase in BBB permeability in comparison with control. By 12 dpi, BBB permeability increase in both cerebra and cerebella of mice infected with GD-SH-01 continued to be enhanced (more than threefold increase of NaF uptake); at this time point, majority of mice infected with GD-SH-01 developed severe neurological signs or died owing to rabies (Figures 2A,B), indicating that BBB was destroyed when rabies reached the final stage (Gnanadurai et al., 2015; Miao et al., 2017). However, the BBB of the mice infected with HEP-Flury recovered generally from transient breakdown, and those infected with rHEP-SH-P kept relative integrity in the course of infection. Overall, the chimeric RABV rHEP-SH-P did not enhance BBB permeability as much as parent strains.

### The Chimeric Strain of RABV Is Less Pathogenic Than Parent Strains

In an effort to examine the pathogenicity of three strains of RABV, we daily recorded the weight changes and clinical signs of infected mice as described in the section “Materials and Methods” (Figure 2). After i.n. inoculation, the majority of mice infected with HEP-Flury and GD-SH-01 strains began to suffer body weight loss and develop clinical signs at 8–10 dpi, though the severity of which was obviously different. Those mice infected with HEP-Flury showed mild neurological signs during 9–12 dpi; meanwhile, the body weight dropped to bottom at 12 dpi (around 80% of original weight); in the following days, the mice recovered from body weight loss and clinical signs gradually. For the GD-SH-01 group, the mice continuously lost body weight (<100% of original weight) from 8 dpi; in the following days, the mice began to develop severe neurological signs till death. These results suggested strongly that GD-SH-01 was able to cause rabies once the virus reached CNS; on the contrary, HEP-Flury caused mild symptoms rather than rabid death. Intriguingly, all of the rHEP-SH-P-infected mice continued to gain body weight (>100% of original weight) until 10 dpi; the mice suffered up to only 5% weight loss and did not develop any symptoms in the following days. These findings confirmed that rHEP-SH-P was an asymptomatic RABV strain and less pathogenic to adult mice than two parent strains.

### TJ Protein Expression Has Been Reduced by Parent Strains of RABV in CNS

**FIGURE 3:**
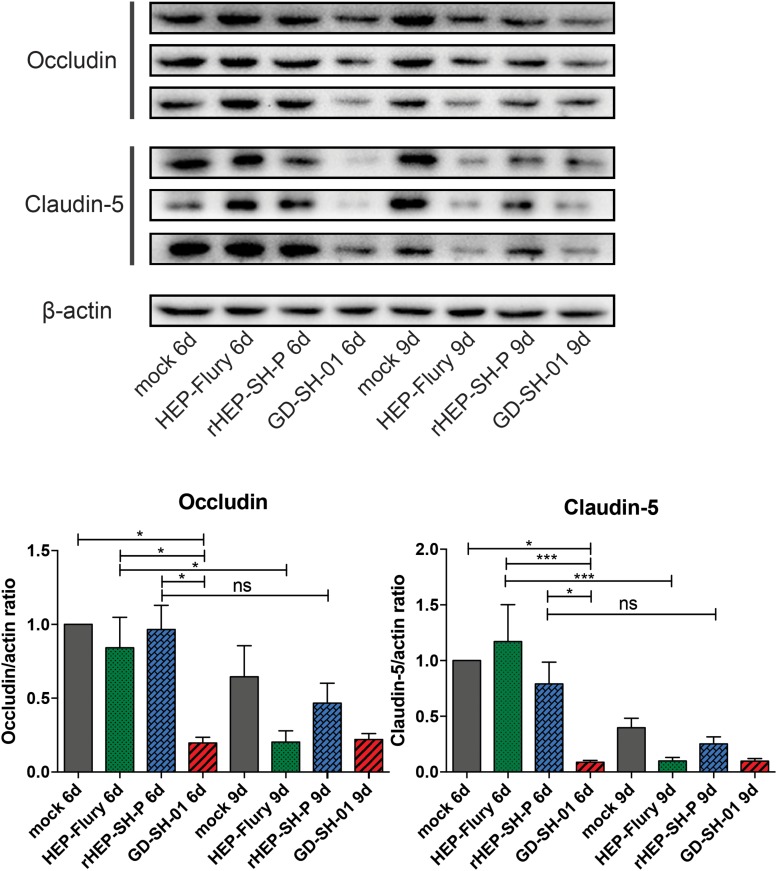
Western blotting analysis of TJ protein (Occludin and Claudin-5) at 6 or 9 dpi in the brains of adult mice infected with HEP-Flury, GD-SH-01, or rHEP-SH-P, and culture medium for control. The expression levels of TJ proteins were analyzed by quantifying IODs using Image J v1.51. The statistical analyses were performed using one-way ANOVA; significant differences between groups were denoted by *p < 0.05 and ***p < 0.001. Data are means ± SEM of results from three independent experiments.

To study the mechanisms of RABVs that enhanced the BBB permeability, the expression levels of TJ proteins (Occludin and Claudin-5) in brains of infected mice were evaluated using Western blotting. According to Figure 3, both Occludin and Claudin-5 signals were significantly weaker in brains infected with GD-SH-01 compared to that of the HEP-Flury and rHEP-SH-P group at 6 dpi, and the expression levels were maintained fairly low till 9 dpi. The significant reductions of TJ protein expression were found in brains infected with HEP-Flury at 9 dpi in comparison with those at 6 dpi. Although the IODs of TJ protein staining were slightly weaker in the rHEP-SH-P group at 9 dpi in comparison with the mock infection group, there was no significant reduction of TJ protein expression observed from 6 to 9 dpi (*p* > 0.05). The pattern of these two proteins in the rHEP-SH-P group was similar to that in mock-infected mice. These findings indicated that RABV strains HEP-Flury and GD-SH-01 broke down the BBB by downregulating TJ protein expression, while rHEP-SH-P maintained the BBB integrity with poor capacity to downregulating TJ protein expression.

### The Chimeric Strain of RABV Induces Less Infiltration of Immune Cells Into CNS Than Parent Strains

**FIGURE 4:**
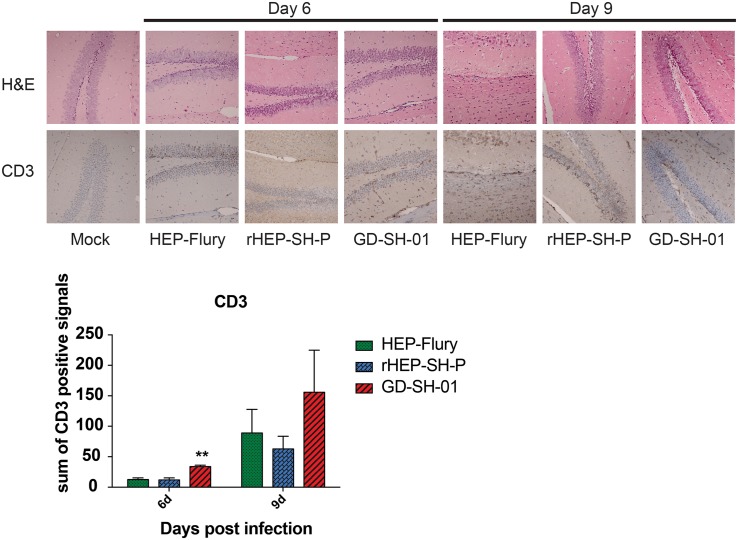
Detection of inflammatory CD3+ T cells in brains of mice. Female KM mice were infected with 10^5^ FFU of HEP-Flury, GD-SH-01, or rHEP-SH-P, and culture medium for control; the fixed brains were subjected to HE staining for histopathology or antibody for IHC. Brain tissues were magnified 400× under microscope. CD3-positive signals were calculated as the count of the DAB signals and labeled with asterisks indicating significant difference compared with other groups, as calculated using the one-way ANOVA (**p < 0.01).

Enhancement of BBB permeability facilitates T cell infiltration. In order to observe the infiltration of immune cells into CNS through BBB, brains of infected mice were harvested and stained with anti-CD3 antibody for detecting signals of CD3^+^ T cells (Kuang et al., 2009; Chai et al., 2014). In Figure 4, CD3^+^ T cells were well observed in the brains infected with GD-SH-01 at 6 and 9 dpi; especially at 9 dpi, the number of positive signal of CD3 boosted (>150 positive signals), which indicated that the enhanced BBB permeability of brains infected with GD-SH-01 facilitated infiltration of T cells into CNS. In contrast, less positive signals of CD3^+^ T cell were detected in rHEP-SH-P-infected brains at two indicated days. Although HEP-Flury did not induce obvious T cell infiltration at 9 dpi, the capability to recruit inflammatory cells was found to be little stronger in comparison with that of rHEP-SH-P. Overall, the observations suggested that the capacity of rHEP-SH-P to induce immune cell infiltration into CNS was poorer than parent strains due to lower BBB permeability.

### Wild-Type RABV P Gene Contributed Little to Inflammation Induction

**FIGURE 5:**
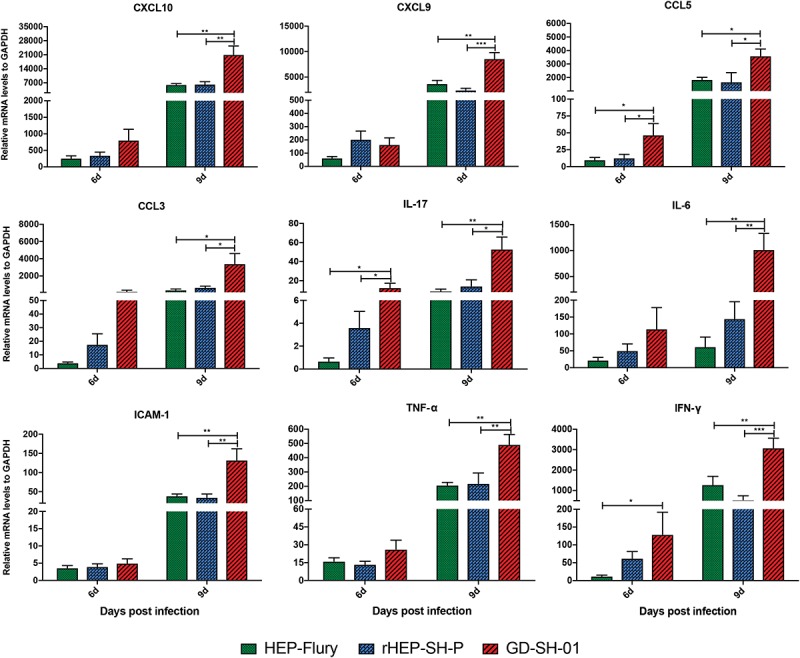
Measurement of cytokines/chemokines expression in brains of infected mice. Female KM mice were infected with 10^5^ FFU of HEP-Flury, GD-SH-01, or rHEP-SH-P, and culture medium for control, and the mRNA levels of cytokines/chemokines were quantitated relative to GAPDH using qRT-PCR at 6 or 9 dpi. The statistical analyses were performed using one-way ANOVA; significant differences between groups were denoted by *p < 0.05; **p < 0.01; ***p < 0.001. Data are means ± SEM of results from three independent experiments.

Previous studies demonstrated that the enhancement of BBB permeability was modulated by cytokines/chemokines (Toborek et al., 2005; Chai et al., 2014). In order to investigate changes of the expression of chemokines (CXCL10, CXCL9, CCL5, and CCL3), interleukins (IL-17 and IL-6), and other cytokines (IFN-γ, TNF-α, and ICAM-1) in CNS, the brains of infected mice were harvested and homogenized to determine the inflammatory response using qRT-PCR at 6 and 9 dpi. According to Figure 5, the expression of CCL5, IL-17, and IFN-γ was significantly higher in brains infected with GD-SH-01 than HEP-Flury and rHEP-SH-P at 6 dpi. By 9 dpi, the expression of all cytokines/chemokines was upregulated; however, GD-SH-01 induced stronger inflammatory responses than HEP-Flury and rHEP-SH-P, and the capacity of rHEP-SH-P to induce cytokines/chemokines was similar to that of HEP-Flury. In particular, the great increase of CXCL10 expression played as the initiator of the cascade of the signaling pathway (Gnanadurai and Fu, 2016). The expression of other cytokines (IFN-γ, TNF-α, and ICAM-1) involving BBB breakdown well positively supported the observations. These findings indicated that GD-SH-01 induced strong inflammatory response in the final stage, which resulted in breakdown of BBB, whereas rHEP-SH-P induced relatively mild inflammatory response to keep BBB integrity.

### Suppressed Wild-Type RABV P Gene Is Responsible for Limited Viral Replication

**FIGURE 6:**
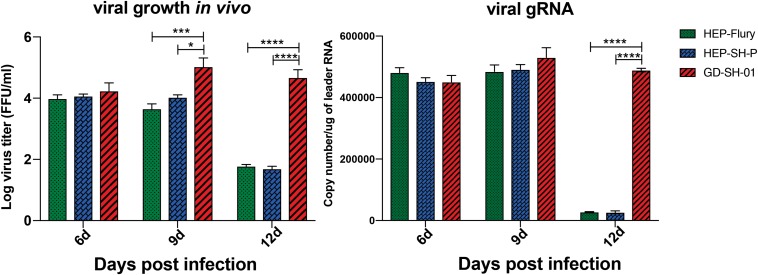
Viral growth kinetics of the three RABVs **(Left)** and viral genomic RNA level *in vivo* **(Right)**. The RABV-infected brains were harvested to isolate the virus and determine the viral titers, and the copy number of genomic RNA was evaluated and standardized by RABV genome plasmid using qRT-PCR. The statistical analyses were performed using one-way ANOVA; significant differences between groups were denoted by *p < 0.05; *** *p* < 0.001; ****p < 0.0001. Data are means ± SEM of results from three independent experiments.

Boost of virus *in vivo* contributes to the inflammatory responses. To provide insights into mechanism by which RABV caused changes of inflammatory responses, viral growth *in vivo* and viral mRNA copy were determined. From viral growth *in vivo* (Figure 6), we found that the titers of GD-SH-01 were significantly higher than HEP-Flury and rHEP-SH-P at 9 and 12 dpi, at which BBB permeability of brains infected with GD-SH-01 was enhanced greatly. In accordance to transcription of viral gRNA, the level of GD-SH-01 gRNA at 12 dpi was significantly higher than that of HEP-Flury and rHEP-SH-P. These data suggested that the stronger replication of GD-SH-01 induced fatal inflammatory responses *in vivo*.

**FIGURE 7:**
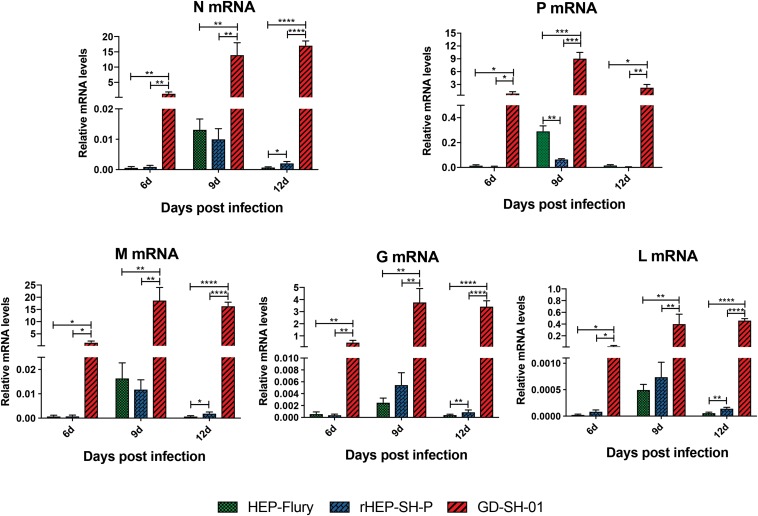
Transcription level of five RABV structural genes *in vivo*. Relative transcription level of structural gene was normalized by GAPDH using qRT-PCR. The statistical analyses were performed using one-way ANOVA, significant differences between groups were denoted by *p < 0.05; **p < 0.01; ***p < 0.001; ****p < 0.0001. Data are means ± SEM of results from three independent experiments.

During the course of infection, the transcription of viral structural genes was detected using qRT-PCR. Interestingly, only P gene expression of rHEP-SH-P was found to be suppressed significantly compared to HEP-Flury during the course of infection (Figure 7); that of other genes (N, M, G, and L) was not obviously different between rHEP-SH-P and HEP-Flury, but all greatly lower than GD-SH-01. Thus, it was obvious that the expression of wt RABV P gene was potentially suppressed, whereas due to the optimal ratio of structural genes (Mei et al., 2017), we observed higher expression level of P gene in GD-SH-01. The limited P gene of rHEP-SH-P hardly facilitated the viral proliferation *in vivo*, which resulted in less activation of inflammatory response.

## Discussion

Our previous study indicated that recombinant rHEP-SH-P that carries the P gene of GD-SH-01 induced significantly greater apoptosis compared with parent strains (Tian et al., 2017a). To further elucidate the role of the wt P gene, the effect of the chimeric RABV rHEP-SH-P on BBB permeability, pathogenicity, and viral replication *in vivo* was evaluated. Our results demonstrated that rHEP-SH-P showed significant lower pathogenicity in mice compared to GD-SH-01, which was consistent with previous studies that show that G protein mainly contributes to the pathogenicity of RABV, rather than P protein (Dietzschold et al., 1983; Ito et al., 2001; Pulmanausahakul et al., 2008), whereas the P gene facilitated viral pathogenesis, as previously reported that P gene-deficient RABV strain was apathogenic to host (Morimoto et al., 2005). Our previous study demonstrated that the lower expression of P gene resulted in weaker pathogenic outcomes (Mei et al., 2017). In the present study, despite that the G gene expression of HEP-Flury and rHEP-SH-P was not significantly different, rHEP-SH-P bearing suppressed P gene was less pathogenic than HEP-Flury.

As a physical and physiological barrier between peripheral circulation and CNS, the BBB inhibits the entry of cells or molecules into CNS by the cell–cell TJs. The TJ complexes consist of numerous families of transmembrane protein; Occludin and Claudins are rarely the two representative members among them (Spindler and Hsu, 2012). The downregulation of Occludin and Claudin-5 was not aligned well to the breakdown of BBB in the GD-SH-01 group at 6 dpi, which further indicated that BBB was an integrated physiological complex whose structure was maintained by various factors (Abbott et al., 2010). Furthermore, the expression of some chemokines highly elevated by GD-SH-01 at 6 dpi caused Occludin and Claudin degradation, whereas it might not be sufficient to cause the BBB breakdown completely yet. Thereafter, the degradation of TJ protein in the HEP-Flury and GD-SH-01 group at 9 dpi was well reflected by enhancement of BBB permeability, while the BBB permeability of the rHEP-SH-P group was maintained at a fairly low level, which was also supported by the expression of TJ protein in Figure 3. HEP-Flury and GD-SH-01 are capable of enhancing BBB permeability by degrading TJ proteins, while rHEP-SH-P failed.

The enhancement of BBB permeability facilitated the entry of immune effectors (including immune cells and cytokines/chemokines) into the CNS to eliminate viruses (Roy et al., 2007; Huang et al., 2014). Figure 4 shows that more CD3^+^ T cells infiltrated into the CNS through the permeable BBB in the HEP-Flury and GD-SH-01 group at 9 dpi, when BBB permeability of the rHEP-SH-P group was fairly low. However, as a pathogenic strain, GD-SH-01 caused irreversible breakdown of BBB at the final stage of rabies as well as other wt RABV strains, which is totally different from transient enhancement of BBB permeability by attenuated RABV strains. Actually, we detected strongly elevated expression of cytokines and chemokines in mice brains infected with GD-SH-01 at 6 dpi, which indicated that the mice got fatal encephalitis, and the infected mice did not recover from rabies till it died at 12 dpi (Gnanadurai et al., 2015; Miao et al., 2017). However, the brains infected with rHEP-SH-P presented a relatively low level of T cells and cytokine expression from day 6 due to the less permeability of BBB. The inflammatory component of the antiviral response has pathological consequences to rabies, and elements of immune response promote the replication and spread of virus (Hooper, 2005; Zhao et al., 2009). Therefore, the boost of GD-SH-01 *in vivo* leads to lethal outcomes despite the breakdown of BBB. Enhancement of BBB permeability caused by rHEP-SH-P was strongly attenuated, which was not beneficial for RABV clearance based on previous studies. As a result, there must be other elements involved in attenuating the pathogenicity of rHEP-SH-P.

The RNP is a key role in the viral assembly and replication; RABV P protein serves as a component of RNP affecting viral replication indirectly. Actually, the formation of RNP was influenced by the optimal ratio of N:P:L (Pattnaik and Wertz, 1990; Mei et al., 2017), which was reflected by the suppressed N and L expression in the current study and suggested that the low level of P gene expression limited the viral replication by obstructing the formation of RNP. A previous study suggested the poor proliferative capacity of rHEP-SH-P *in vitro* (Tian et al., 2017b); in the current study, we observed relatively lower viral growth of rHEP-SH-P *in vivo*, which was due to the suppressed P gene. Furthermore, we assume that the poor replication capacity of rHEP-SH-P hampers the viral infection and pathogenicity in hosts.

Inflammation was necessary to clear RABV in CNS despite its pathogenic outcomes. It has been reported that the neurons infected with attenuated RABV secrete CXCL10, which mediates the recruitment of T cells into CNS and differentiates to Th17 cells and upregulates IFN-γ expression, and further boosts the induction of CXCL10 and IL-17-secreting Th17 cells, altering the TJ proteins (Huppert et al., 2010; Chai et al., 2015; Gnanadurai and Fu, 2016). Thus, CXCL10 plays as an initiator of the cascade. In addition, TNF-α has been shown to induce BBB disruption as the result of decreased trans-endothelial electrical resistance (TEER) of brain microvascular ECs (BMECs) (Dickstein et al., 2000). However, some scientists reported that intracarotid injection of TNF-α decreased BBB permeability in rats (Petito et al., 1999). Although the effect of TNF-α on BBB remains unclear, CXCL10 can be stimulated by TNF-α (Ohmori and Hamilton, 1995). Therefore, upregulated expression of these cytokines/chemokines in brains of mice infected with GD-SH-01 causes BBB breakdown. In the current study, GD-SH-01 induced a high level of chemokines in late stage of infection due to its strong replication *in vivo*, which facilitated CXCL10 to recruit T cells into CNS and initiate the breakdown of BBB permeability. In contrast, because of poor replication in the whole course of infection, rHEP-SH-P induced mild inflammation and was less pathogenic than GD-SH-01. Although no significant difference was observed between HEP-Flury and rHEP-SH-P in cytokine/chemokine expression to explain the BBB permeability changes, we assume that the limited P gene was somehow related with viral pathogenicity and/or other BBB breakdown pathway based on the fact that rHEP-SH-P and HEP-Flury share a genomic backbone (Figure 7); after all, the virus properties are attributed to the five genes. However, we do believe that the wt-RABV P gene potentially hampers viral replication, which is responsible for inflammation induction based on our findings.

## Conclusion

In conclusion, less enhancement of BBB and inflammatory response was observed in mice infected with rHEP-SH-P than parent strains, and this chimeric RABV strain caused less weight loss of adult mice and milder symptoms than parent strains. Meanwhile, the limited replication of rHEP-SH-P and suppressed P gene expression were detected, which suggested that RABV P gene played as a potential determinant that hampered the viral invasion and RABV proliferation to realize immune escape. One of the limitations of this study is the failure of evaluating HEP-Flury P gene *in vivo* in the genomic backbone of GD-SH-01. However, to our knowledge, the present data revealed the relationship between wt RABV genetic function and pathogenetic outcomes. The manner that RABV P gene affected the viral pathogenesis in collaboration with G gene is unclear so far; thus, more research especially on intra-genomic interaction is required in the future to further reveal the global mechanism of RABV P gene in viral replication and pathogenicity. Moreover, as mentioned above, BBB is an integrated complex, and future studies on other pathways of degrading BBB integrity could be a foundation for therapeutic strategy of rabies.

## Data Availability Statement

The datasets generated for this study are available on request to the corresponding author.

## Ethics Statement

All procedures involving animals were approved by the Ethics Committee for Animal Experiments at South China Agricultural University. All animal experiments were carried out in strict accordance with international, national, and institutional guidelines for the use and care of animals.

## Author Contributions

XG and TL contributed to the study design. TL, BZ, RF, YW, MeM, HJ, and YC performed the research. TL, QT, MiM, and YL contributed to the data analysis. TL wrote the original draft of the manuscript. XG and JL reviewed and edited the manuscript. All the authors approved the manuscript for publication.

## Conflict of Interest

The authors declare that the research was conducted in the absence of any commercial or financial relationships that could be construed as a potential conflict of interest.
