# Supplementary material for: Phosphoprotein Gene of Wild-Type Rabies Virus Plays a Role in Limiting Viral Pathogenicity and Lowering the Enhancement of BBB Permeability
Source: Front Microbiol. 2020 Feb 20;11:109. doi: 10.3389/fmicb.2020.00109 (PMC7045047; doi:10.3389/fmicb.2020.00109)
Supplement: Supplementary file 1 [file Table_1.DOCX]

**TableS1.** Primer pairs used in this study.

| Primer name | Sequence (5’-3’) | Length of product (bp) |
| --- | --- | --- |
| CXCL10-F | TACTGTAAGCTATGTGGAGGTGCG | 106 |
| CXCL10-R | AACTTAGAACTGACGAGCCTGAGC |  |
| CXCL9-F | AAACCTGCCTAGATCCGGAC | 99 |
| CXCL9-R | GTTTTTTCCCCCTCTTTTGC |  |
| CCL5-F | CCTCACCATCATCCTCACTG | 99 |
| CCL5-R | AGGGAGAGGTAGGCAAAGCA |  |
| CCL3-F | AAGCTCACCCTCTGTCACCTG | 100 |
| CCL3-R | CTGAGAAGACTTGGTTGCAGAGTG |  |
| IFN-γ-F | AGCAACAACATAAGCGTCATT | 100 |
| IFN-γ-R | CCTCAAACTTGGCAATACTCA |  |
| TNF-α-F | AGGTTCTCTTCAAGGGACAAG | 103 |
| TNF-α-R | GCAGAGAGGAGGTTGACTTTC |  |
| IL6-F | GGCATAACGCACTAGGTTT | 117 |
| IL6-R | GCTGGAGTCACAGAAGGAG |  |
| IL17-F | TCCTCCAGAATGTGAAGGTCAACC | 134 |
| IL17-R | TCTATCAGGGTCTTCATTGCGGTG |  |
| ICAM1-F | CTGCAGACGGAAGGCAGATGGT | 99 |
| ICAM1-R | GAGCTAAAGGCATGGCACACGTA |  |
| HEP-SH-N_F | GGAACCTACGACATGTTTTTC | 92 |
| HEP-SH-N_R | CCTGAGCAGTCTTCATAAGC |  |
| HEP-SH-P_F | ATCCGAGTGCAATCAGAGCC | 104 |
| HEP-SH-P_R | TCCCTGGAGATGAGCCTGATT |  |
| HEP-SH-M_F | TAGTGAAAAACTGTAGGGATGA | 112 |
| HEP-SH-M_R | AGTTCTTTCAGCGGGACATAT |  |
| HEP-SH-G_F | TGCTCAGGAATAACGGTGTC | 128 |
| HEP-SH-G_R | GATGCTCTCTTCCCTCTGCT |  |
| HEP-SH-L_F | GATCCGGGAGAGGTTTATGAT | 101 |
| HEP-SH-L_R | TAGTCAGAGTTCCTCAAGATGTT |  |
| RABV gRNA-F | AGAAGAAGCAGACATCGTCAGTTG | 98 |
| RABV gRNA-R | GGAGACCACCTGATTATTGACTTTGA |  |
| GAPDH-F | CGTCCCGTAGACAAAATGGT | 110 |
| GAPDH-R | TTGATGGCAACAATCTCCAC |  |
